# Climate‐induced distribution dynamics of *Plebeia flavocincta*, a stingless bee from Brazilian tropical dry forests

**DOI:** 10.1002/ece3.6674

**Published:** 2020-08-20

**Authors:** Ulysses Madureira Maia, Leonardo de Sousa Miranda, Airton Torres Carvalho, Vera Lucia Imperatriz‐Fonseca, Guilherme Corrêa de Oliveira, Tereza Cristina Giannini

**Affiliations:** ^1^ Instituto de Ciências Biológicas Universidade Federal do Pará Belém Brazil; ^2^ Instituto Tecnológico Vale Belém Brazil; ^3^ Unidade Acadêmica de Serra Talhada Universidade Federal Rural do Pernambuco Serra Talhada Brazil; ^4^ Instituto de Biociências Universidade de São Paulo São Paulo Brazil

**Keywords:** availability, Caatinga, expansion, geographic distribution, Meliponin, pollinator

## Abstract

**Aim:**

The objective of this study is to estimate the current potential geographic distribution of *Plebeia flavocincta* and to evaluate the influence of climate on the dynamics of suitable habitat availability in the past and in the future.

**Location:**

Northeast region of Brazil and dry forest areas.

**Methods:**

The habitat suitability modeling was based on two algorithms, two global circulation models, and six different scenarios. We used this tool to estimate the areas of occurrence in the past (Last Interglacial and Last Glacial Maximum), in the present, and in the future (years 2050 and 2070).

**Results:**

According to the models, *P. flavocincta* had great dynamics in the availability of suitable habitats with periods of retraction and expansion of these areas in the past. Our results suggest that this taxon may benefit in terms of climate suitability gain in Northeast Brazil in the future. In addition, we identified high‐altitude areas and the eastern coast as climatically stable.

**Conclusion:**

The information provided can be used by decision makers to support actions toward protecting and sustainably managing this taxon. Protection measures for this taxon are particularly important because this insect contributes to the local flora and, although our results indicate that the climate may favor this taxon, other factors can negatively affect it, such as high levels of habitat loss due to anthropogenic activities.

## Graphical abstract

We analyzed the influence of climate on the dynamics of suitable areas and presented priority areas for the conservation of a stingless bee, an important pollinator in the endemic and threatened dry forest biome distributed across northeastern Brazil. Yellow areas represent locations indicated by all scenarios as suitable for the species considering (a) past, present, future and (b) present and future climate conditions.

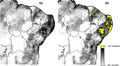

## 1 INTRODUCTION

**FIGURE 1:**
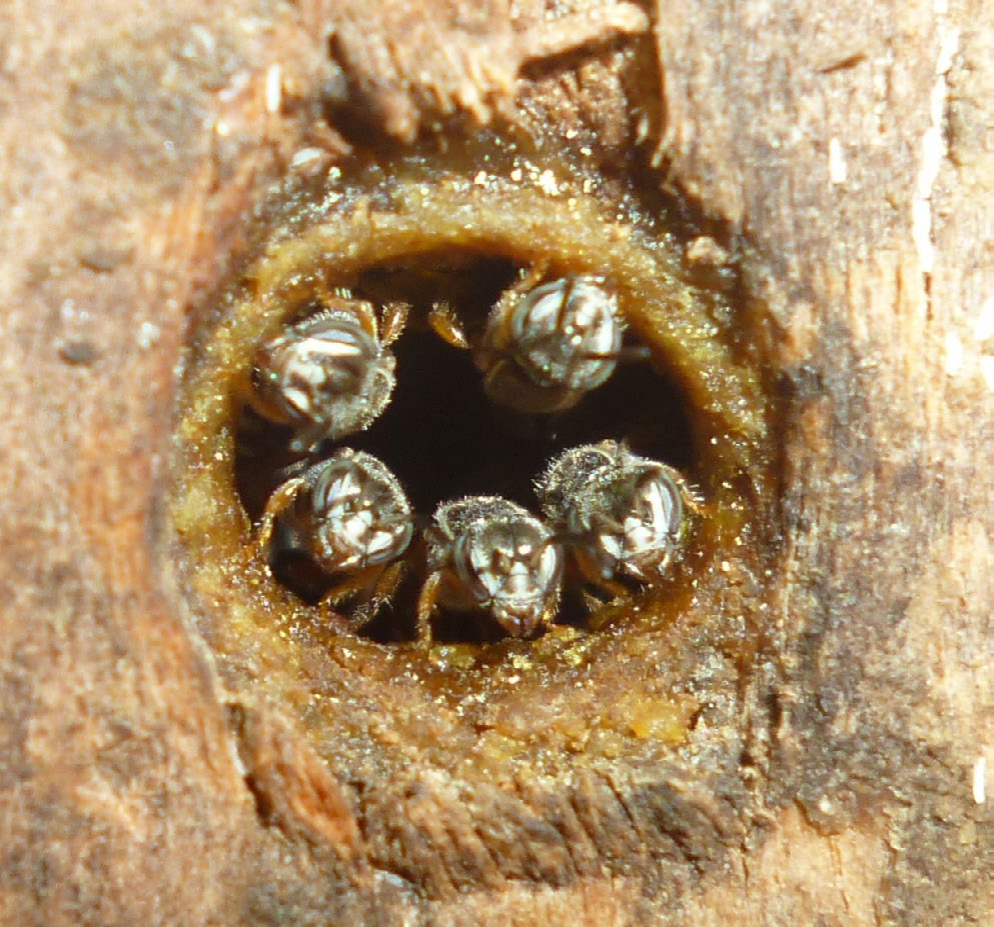
Workers of *Plebeia flavocincta* at the entrance of the nest in a rational wooden hive

The genus *Plebeia* Schwarz, 1938, is a very diverse group that is widely distributed and can be found from northern Mexico to Central Argentina (Silveira, Melo, & Almeida, 2002). In Brazil, there are 19 species (Pedro, 2014) of the 40 recorded for this genus (Camargo & Pedro, 2013). Among these species, *Plebeia flavocincta* (Cockerell, 1912) is a small bee seemingly well adapted to the climatic conditions of Northeast Brazil (Figure 1). This taxon occurs in tropical dry forests (Zanella & Martins, 2003) and interacts with approximately 60 plant species (Costa, 2014), and can be considered an important visitor of the native flora.

Tropical dry forests of northeastern Brazil, also known as Caatinga, consist of an ecoregion composed predominantly of xerophilic, woody, thorny, and deciduous vegetation structures (Costa, de Araújo, & Lima‐Verde, 2007; Sampaio, 1995), and a hot and dry climate (Veloso, Rangel‐Filho, & Lima, 1991), extending over more than 750,000 km^2^ (Silva, Barbosa, Leal, & Tabarelli, 2017). The unique climatic conditions of the Caatinga are responsible for the existence of unique biodiversity adaptations, which allow for a large number of species adapted to heat and drought. Caatinga areas are undergoing extensive processes of environmental alteration and deterioration caused by unsustainable resource use (Leal, Tabarelli, & da Silva, 2003; Silva et al., 2017). In addition, there is a high risk of desertification in the Caatinga, resulting from several factors, including human activities and climatic variations (Santana, 2007). Different initiatives have projected future climate change scenarios, with most predicting an increase in temperature and a decrease in precipitation in this region (Marengo, Alves, Beserra, & Lacerda, 2011; Torres, Lapola, & Gamarra, 2017). According to the 5th assessment report of the Intergovernmental Panel on Climate Change (AR5 IPCC 2014), climate scenarios suggest that the main threat to the Caatinga is increasing aridity, which could lead to an intensification and prolongation of the dry seasons. However, at least for endemic birds and reptiles, most species analyzed using habitat suitability models (HSMs) were projected to gain climatically suitable areas in remnants of natural Caatinga vegetation (Oliveira, Araújo, Rangel, Alagador, & Diniz‐Filho, 2012).

There is extensive evidence of a decline in pollinators around the world, with habitat fragmentation/loss and climate change as the main causes of this decline (Potts et al., 2010). Climate change can affect bees on different levels, such as behavior, physiology, quality of the floral environment, population dynamics, and the emergence of new interactions between species and parasites and pathogens (Le Conte & Navajas, 2008). Small bee species are more likely to be affected by changes in climatic conditions because they lose heat faster than larger bees due to their small body size (Oyen & Dillon, 2018). This is supported by Costa (2014) and Silva, Meneses, and Freitas (2019), who observed that *P. flavocincta* prefers to start its flight activity during periods of higher temperatures.

The spatial distribution of *P. flavocincta* is not precisely known, which hinders the development of conservation and management plans for this taxon. Habitat suitability models have been used to both evaluate the niche dynamics of a species and determine the most important environmental variables that affect its potential distribution (Pearman, Guisan, Broennimann, & Randin, 2008). The applications of this tool for conservation purposes are based on the projection of climatically suitable areas in the present and in the future or on the investigation of the potential historical distribution of a species. This tool is increasingly used to predict the effects of climate change over time on species distribution (Franklin, 2010).

The objective of this study is to estimate the current potential geographic distribution of *P. flavocincta* and evaluate the influence of climate on the dynamics of suitable habitat availability in the past and in the future. We focused on occurrence records of what is now taxonomically recognized as *P. flavocincta* and asked how climatic fluctuations could affect the distribution dynamics of the taxon in the past and in the future.

## 2 METHODS

### 2.1 Study area

**FIGURE 2:**
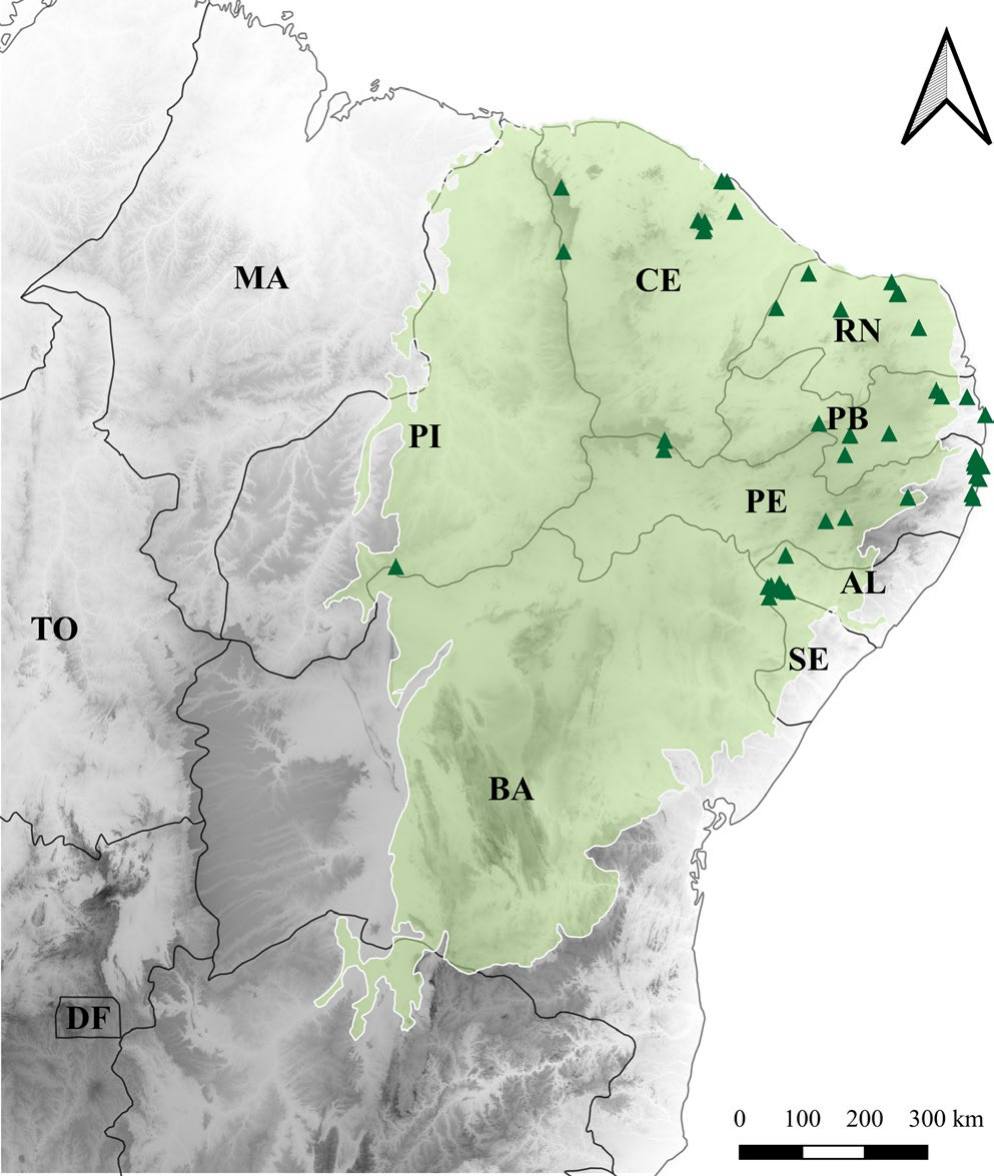
Study area used for model projections. The thick black line delimits the Northeast region of Brazil; the green area delimits the Caatinga domain; the gray gradient represents altitude—darker colors, higher altitude; rivers are in blue; and green triangles are the occurrence records of *Plebeia flavocincta* used for this study

Northeast Brazil is strongly influenced by precipitation scarcity and unpredictability (Bronstert, Carrera, Kabat, & Lütkemeier, 2005). The Caatinga constitutes most of this area (Figure 2), which has a semiarid climate with precipitation of 500–800 mm/year (Velloso, Sampaio, & Pareyn, 2002). Precipitation is strongly seasonal (during only 3–4 months of the year, between December and March) and has a high interannual variability, which leads to severe recurrent and irregular droughts (Brito et al., 2018).

### 2.2 Georeferenced occurrences

We gathered data from the digital platforms Global Biodiversity Information Facility (GBIF; www.gbif.org, accessed in February 2019), SPECIESLINK (www.splink.org.br, accessed in February 2019), and complementary data collected in the field, totaling 615 records. We verified the information about who identified each specimen in each record found in the digital databases, and we kept only entries made by specialists. We also applied a filter to remove duplicates and nongeoreferenced data. Thus, our final database contained 52 occurrence records (30 from digital database and 22 from our field collections) for *P. flavocincta* in seven Brazilian states (Figure 2). Most of the points are located in Caatinga areas.

### 2.3 Climate data

The species distribution models were calibrated using current climate data from the WorldClim database (www.worldclim.org, accessed in February 2019) with a resolution of ~1 km^2^. We tested which of the 20 variables were most strongly correlated with each other (*r* > .75) through the "removeCollinearity" function of the *virtualspecies* package in R (Leroy, Meynard, Bellard, & Courchamp, 2016; R Development Core Team, 2019) and selected nine bioclimatic variables: mean diurnal range (bio 2), isothermality (bio 3), temperature seasonality (bio 4), maximum temperature of the warmest month (bio 5), annual precipitation (bio 12), precipitation seasonality (bio 15), precipitation of the warmest quarter (bio 18), precipitation of the coldest quarter (bio 19), and altitude.

### 2.4 Habitat suitability model

The HSM was based on records of presence and pseudoabsence. We generated three sets of pseudoabsence data containing ten times the number of randomly distributed presence data points (Chefaoui & Lobo, 2008). We used two algorithms: generalized linear model (GLM) (McCullagh & Nelder, 1989) and maximum entropy (MAXENT) (Phillips, Anderson, & Schapire, 2006), which have been commonly used for generating robust responses (Li & Wang, 2013). The databases were partitioned, with 80% of the original data used for model calibration and 20% for evaluation. This procedure was repeated 10 times to ensure that the predictive accuracy of the model was not affected by the random partition strategy. In addition, we evaluated the quality of these runs according to two different metrics: true skill statistics (TSS, Allouche, Tsoar, & Kadmon, 2006) and receiver operating characteristic curve (ROC; Phillips et al., 2006). Lastly, we projected the probabilities of climatic habitat suitability for the taxon in the present and under past climatic conditions (Last Interglacial—LIG, ~ 120,000 years ago; Last Glacial Maximum—LGM, ~21,000 years ago) and for four scenarios (years 2050 and 2070 and representative concentration pathways [RCPs] 4.5 and 8.5) in our study site (Figure 2). For the LGM and future scenarios, we also used the conditions estimated by two global circulation models (GCMs): MIROC and CCSM4. The results of the different runs for each projection were consolidated (ensembles) using the committee averaging method, which represents a measure of agreement with the results (Thuiller, Lafourcade, Engler, & Araújo, 2009). True skill statistics values lower than 0.5 indicate that the model's performance is not better than a random model. Thus, for the ensemble construction stage, models with TSS values below 0.7 were removed. Lastly, these maps were binarized using the cutoff value (GLM = 474 and MAXENT = 374) that maximizes the sensitivity and specificity of the models. All analyses were performed using the BIOMOD2 package (Thuiller et al., 2009) in R (R Development Core Team, 2019), and the maps were manipulated using QGIS software (QGIS Development Team, 2018).

## 3 RESULTS

### 3.1 Model performance and environmental variables

**FIGURE 3:**
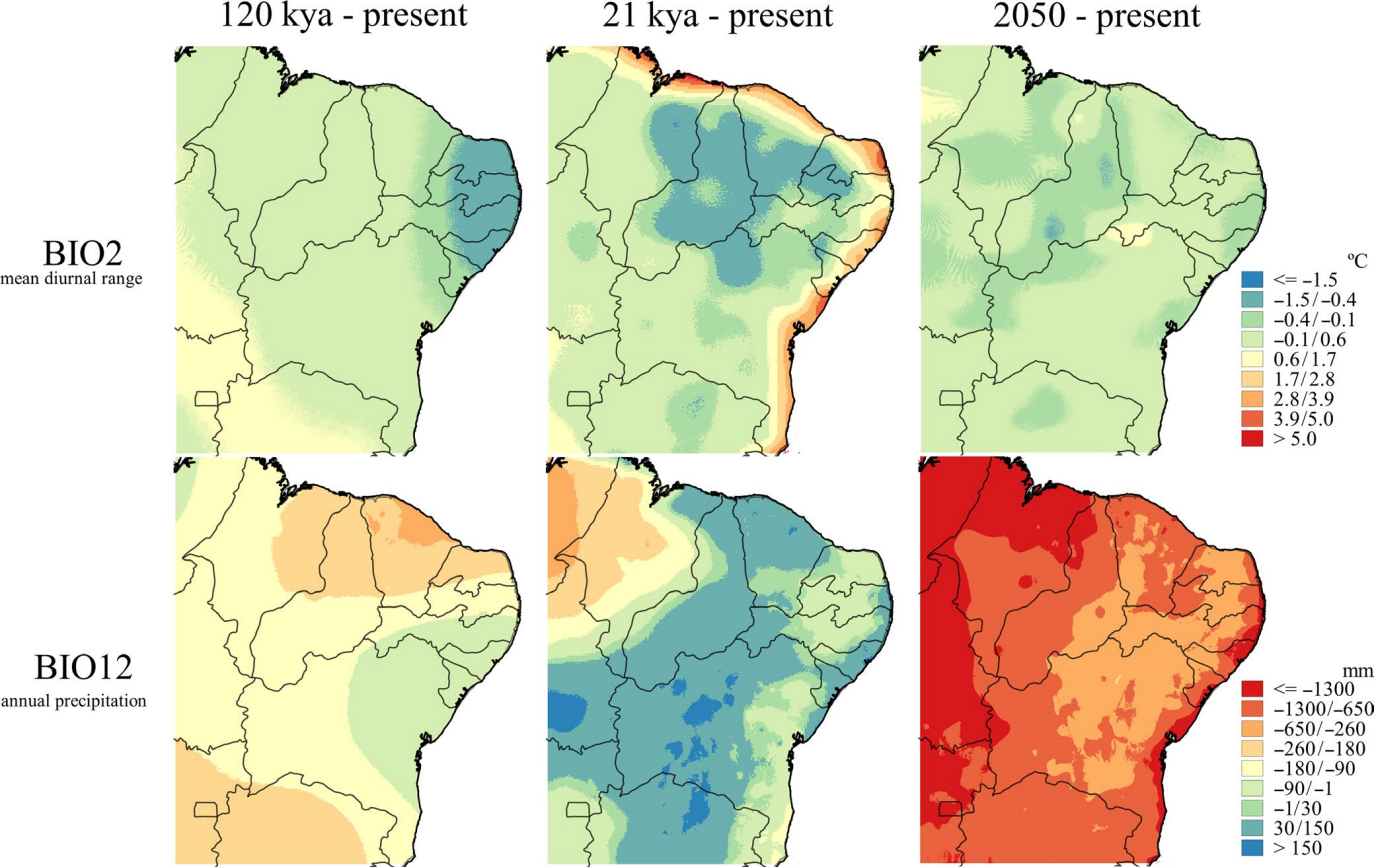
Differences in values between scenarios and the present for mean diurnal range (bio 2) and annual precipitation (bio 12). Warmer colors (red) represent areas with greater diurnal temperature range and lower annual precipitation compared to the present

In general, the different runs of the models exhibited high evaluation scores, that is, values that reflect the quality of the models (TSS and ROC > 0.8). The mean diurnal range (bio 2) and annual precipitation (bio 12) were the most important environmental variables for HSMs (Figure 3). According to the models, in the LIG (~120 kya), the eastern coast of the Northeast region was relatively colder and drier compared to the current mean diurnal temperature range and annual precipitation. For the LGM (~21 kya), this same area had higher mean temperature ranges compared to the present; in addition, the central Caatinga had higher annual precipitation rates.

Figure 3 also shows the projections of the bio 2 and bio 12 variables for only the closest business‐as‐usual scenario (2050, RCP 8.5). For the mean diurnal temperature range (bio 2), there are no major changes in the entire region relative to the present, but there is a considerable increase in temperature (≈0.9°C) to the north of Bahia and southwest of Pernambuco, near one of the driest sites of the semiarid region. For annual precipitation (bio 12), Northeast Brazil will become even drier compared to today, as it was at 120 kya, mainly along the entire eastern coast, where the precipitation rate may decrease >600 mm.

### 3.2 Species distribution over time

**FIGURE 4:**
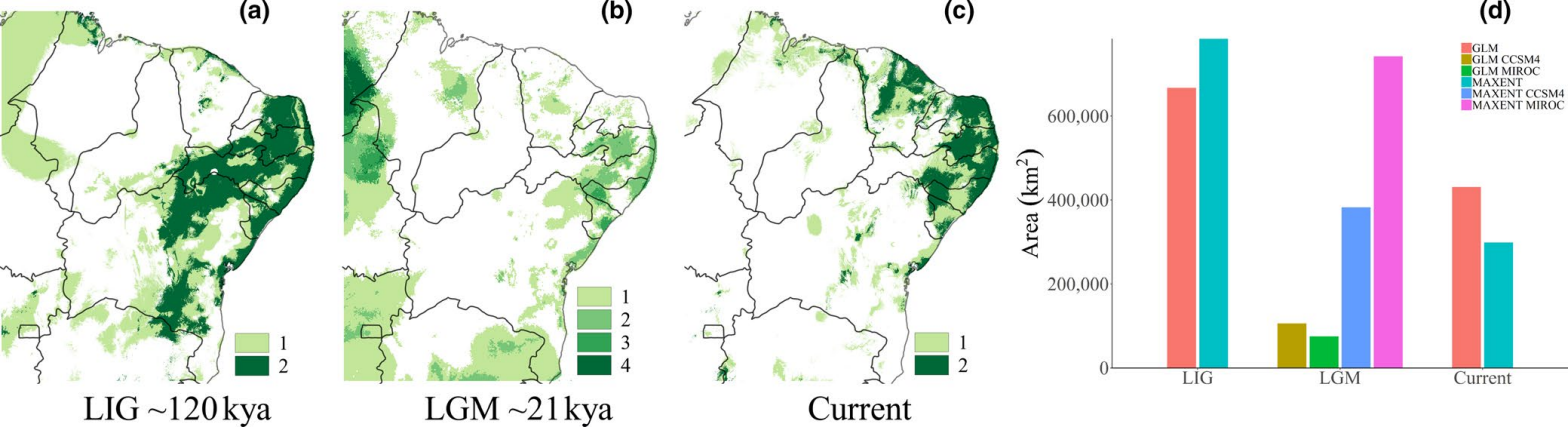
Potential distribution maps of suitable habitats for *Plebeia flavocincta* in the Last Interglacial (a), Last Glacial Maximum (b), and in the present (c). The green tones represent areas where more than one model (algorithms + circulation models) overlaps. Graph (d) shows the size of the estimated area in each model, calculated from the binarized consensus maps by multiplying the number of pixels by the pixel size (~1 km)

According to our models, the distribution of suitable habitats for *P. flavocincta* during the LIG was greater than the current potential distribution, occurring in the central Caatinga region and almost in the entire eastern coast of the Northeast region (Figure 4a). The distribution of *P. flavocincta* in the LGM varies greatly between the global circulation models and algorithms, but in all cases, there was a large retraction mainly in the central–northern portion of Bahia and Rio Grande do Norte (Figure 4b). Interestingly, the projections indicate suitable locations for the occurrence of *P. flavocincta* during the LGM at the extreme west and south of the study site, where the taxon is known not to occur. In fact, the LGM may have caused important changes in taxon distribution and a clear reduction in area (Figure 4b). The projected distribution of *P. flavocincta* in the present day is divided into two highly suitable locations: one in the northeastern study area (in the states of Rio Grande do Norte, Paraíba, Pernambuco, Alagoas, and Sergipe) in areas of Caatinga and Atlantic Forest, and another location in the state of Ceará in high‐altitude areas (≈700 m, Serra da Ibiapaba, Serra das Matas, and Maciço de Baturité mountain ranges) and the coast. Thus, the HSM suggests a large extension of areas with suitable habitats in the LIG, followed by a retraction in the LGM with subsequent expansion of these suitable habitat areas for the present time.

**FIGURE 5:**
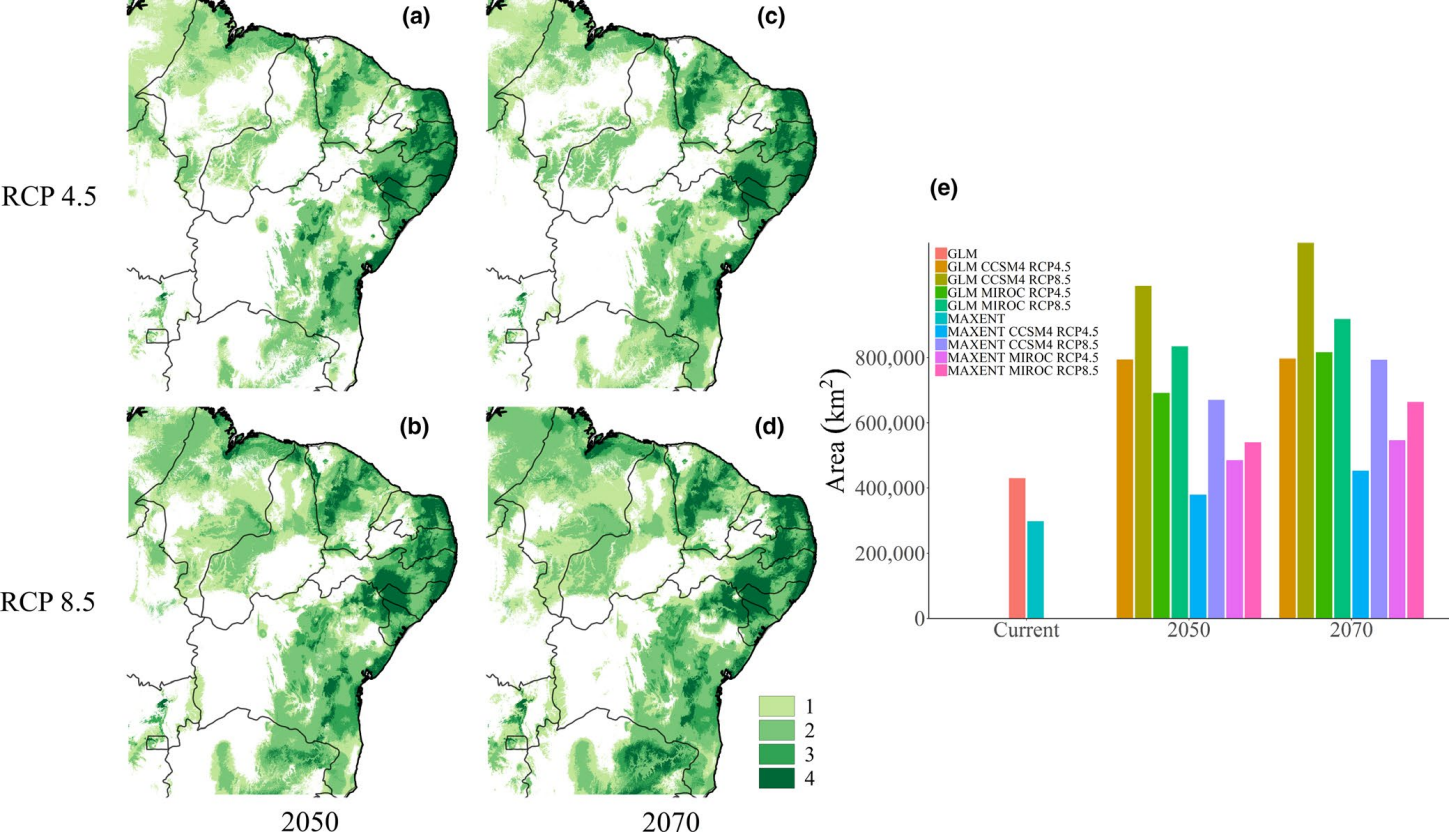
Potential distribution maps of suitable habitats for *Plebeia flavocincta* in the future: (a) 2050 RCP 4.5; (b) 2050 RCP 8.5; (c) 2070 RCP 4.5; and (d) 2070 RCP 8.5. Green tones represent areas where more than one model (algorithms + circulation models) overlaps. Graph (e) shows the size of the estimated area in each model, calculated from the binarized consensus maps by multiplying the pixel number by the pixel size (~1 km)

Our models indicate that the taxon will potentially gain more suitable areas successively over time (i.e., from the present to 2050 and to 2070) in the central region (in the states of Maranhão, Piauí, Bahia, and northernmost of Minas Gerais) and throughout the eastern coast of the study site regardless of the scenarios (RCP 4.5 and RCP 8.5). However, in the central region, between the states of Bahia, Ceará, Paraíba, Pernambuco, Piauí, and Rio Grande do Norte, there is a large range of areas with lower (or no) suitability for both future scenarios analyzed (Figure 5).

### 3.3 Stability of the areas

**FIGURE 6:**
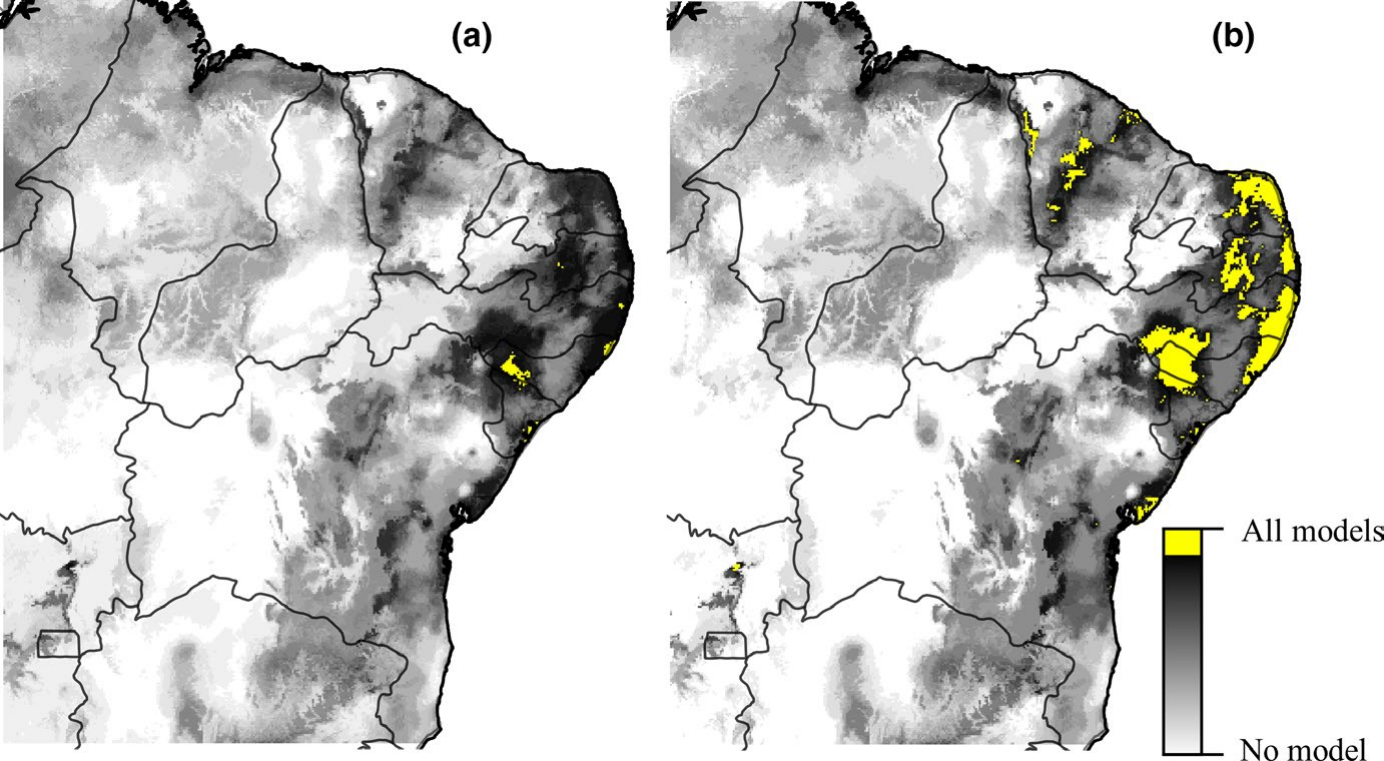
Areas of potential climatic stability resulting from the overlapping of suitability maps for *Plebeia flavocincta*. The areas in yellow represent locations indicated by all scenarios/models as suitable for the species. (a) past, present, and future, “All models” = 24; (b) present and future only, “All models” = 18

Consensus maps of all scenarios were overlapped to determine common areas that represented potential occurrence regardless of the scenario (which we considered climatically stable and, therefore, we call refuge areas; Figure 6). When analyzed with all models (past, present, and future, totaling 24 models), the refuge areas are found in a low region of the São Francisco River Valley, between the states of Sergipe and Alagoas, on the eastern coast (in the states of Pernambuco, Alagoas, and Sergipe) and a small transition area of Atlantic Forest and Caatinga of Paraíba (Figure 6a). When only the projection models for the future were analyzed, several climatically suitable areas were also observed in low areas of the São Francisco River Valley, in areas of the Serra da Ibiapaba, Serra da Mata, and Maciço de Baturité mountain ranges (state of Ceará), transition area of Atlantic Forest and Caatinga of Paraíba and along the entire eastern coast, in several states of the Northeast region (Figure 6b).

## 4 DISCUSSION

The distribution dynamics of *P. flavocincta* in the past were intense, as the total area of climatically suitable habitats projected for the oldest period (LIG, ~120,000 years ago) was much larger than the area of climatically suitable habitats projected for the present. Over time, there was a potential retraction in the LGM scenario (LGM, ~21,000 years ago), followed by a subsequent expansion for the current scenario. Regarding the future, the climatically suitable habitat areas for *P. flavocincta* will potentially increase considering the Caatinga and the northeastern coastal areas. A larger area of climatically suitable habitats for the taxon is associated with hot and dry areas.

In addition to being the environmental variables that most contributed to explaining the models, the mean diurnal range (bio 2) and annual precipitation (bio 12) varied greatly over time. The projected locations of suitable habitat for *P. flavocincta* seem to be associated with higher temperatures and lower precipitation rates. Some studies show that bee flight activity may be limited depending on temperature and precipitation rate (Hilário, Ribeiro, & Imperatriz‐Fonseca, 2012; Silva et al., 2019). Due to a small body size, workers lose heat faster and therefore need higher temperatures for foraging activities (Oyen & Dillon, 2018). Researchers have observed that *P. flavocincta* prefers to forage at warmer times of the day (Silva et al., 2019), as do other species of this genus in other regions of Brazil (*Plebeia droryana* (Friese, 1900), *Plebeia emerina* (Friese, 1900), and *Plebeia saiqui* (Friese, 1900); Hilário, Imperatriz‐Fonseca, & Kleinert, 2001). Similarly, in locations with higher precipitation, bee flight activity may decrease (for example, *Plebeia remota* (Holmberg, 1903); Hilário et al., 2012).

Flight activity is directly related to the dispersal ability of a species. Our models projected for the past suggest an intense dynamic in the distribution of suitable habitats with signs of retraction–expansion through which populations potentially became isolated and that may have left genetic signatures. According to paleodistribution and palynological validation models, the potential historical distribution of Caatinga areas was more restricted during the LGM (Werneck, Costa, Colli, Prado, & Sites, 2011). This retraction phase of the Caatinga during the supposedly dry and cold period of the LGM was followed by an expansion with small fluctuations until reaching the current area. This phenomenon may explain the distribution dynamics of *P. flavocincta* during periods of vegetation retraction and expansion. In fact, it is possible to observe variations in *P. flavocincta* populations regarding behavior, worker coloration, and hive morphology (personal observations) that may be associated with this dynamic. However, for another stingless bee species endemic to the region, *Partamona rustica* Pedro & Camargo, 2003, its potential distribution has been continuously expanding since the LIG (Miranda et al., 2016). For this reason, more specific studies on population variability (i.e., wing morphometry and genetic analyses) are needed to confirm these assumptions.

Our results also suggest that *P. flavocincta* will find a larger area of climatically suitable habitat in the 2050s and especially in the 2070s. The greatest gains are found on the northeastern coast and part of the central Caatinga region. These new areas, especially the areas of Serra de Ibiapaba and Serra da Mata (Ceará) and the transition area of Atlantic Forest and Caatinga of Paraíba and Rio Grande do Norte, also coincide largely with the new climatic suitability areas previously predicted for *Melipona subnitida* Ducke, 1910 (Giannini et al., 2017). There is a band with a lower probability of habitat suitability in the most central region of the Caatinga in the states of Ceará, Bahia, Paraíba, Pernambuco, Piauí, and Rio Grande do Norte. Due to the lower number of samples in the central Caatinga region, these results may underestimate the potential distribution of *P. flavocincta* in this area. In turn, this region is known to be susceptible to desertification (Salazar, Nobre, & Oyama, 2007) due to the joint effects of increasing temperatures, reduced precipitation and soil degradation (Darhoh, 1998; Geist & Lambin, 2004; Sivakumar, 2007).

From a climate change perspective, the area size of a domain may change (Salazar et al., 2007), and new climatically suitable areas may emerge, causing *P. flavocincta* to further expand its area of occurrence. However, underlying the spatial structure of climatic impacts is the pressures of human occupation, land degradation, and biological interactions that may prevent the taxon from occupying these areas. Despite covering an area of approximately 10% of the national territory, only 14% of the Caatinga is protected by law as national conservation areas (Gariglio, Sampaio, Cestaro, & Kageyama, 2010; Sparovek, Berndes, Klug, & Barretto, 2010); that is, its biodiversity has been largely ignored by conservation policies (Leal, Silva, Tab arelli, & Lacher, 2005). Many of the new climatically suitable areas for *P. flavocincta* (2050 and 2070) will coincide with Conservation Units (www.mma.gov.br/areas‐protegidas/cadastro‐nacional‐de‐ucs/map). These conservation areas are important to contain or reduce deforestation and allow the taxon to shelter in protected areas.

The refuge areas estimated by our results are located in the mountains of Ceará, in the central portion of Paraíba, on the eastern coast, and in the lower region of the São Francisco River Valley, among the states of Sergipe, Alagoas, and Pernambuco. These refuge areas are sites where climatic changes are less intense, remaining suitable habitats for the taxon over time and, therefore, possibly representing priority conservation areas. In addition to delimiting priority areas, the planting of native species that provide food resources for *P. flavocincta*, such as *Mimosa tenuiflora* (Willd.) Poir. (Fabaceae), may represent an important step for species conservation (Costa, 2014). Such plantings may be combined with the recovery of degraded areas and the creation of ecological corridors, especially in times of climatic change and scarcity of floral resources in the dry season. Recently, das Chagas, Lucas, and de Almeida Vieira (2020) projected a potential reduction in the climatically suitable areas for *M. tenuiflora* when comparing the current and the future (2070) scenarios. The reduction of climatically suitable areas was discussed as potentially causing spatial incompatibility in pollination interactions (Gérard, Vanderplanck, Wood, & Michez, 2020). However, little is known about climate change impact on plant–bee interactions (but see Giannini et al., 2013); moreover, species' ability to adapt and persist is often neglected. It is also necessary to consider that most species of the tribe Meliponini nest in the hollows of living trees (Nogueira‐Neto, 1997) and are dependent on forest to nidify (Giannini et al., 2015, 2017). In areas of Caatinga, *P. flavocincta* prefers to nest in *Cenostigma pyramidale* (Tul.) Gagnon & GP Lewis (Fabaceae), a native tree species popularly known as *catingueira* and widely used for nesting by other stingless bee species (Martins, Laurino, Koedam, & Fonseca, 2004).

## 5 CONCLUSION

Our results contribute to the interpretation of biogeographic scenarios in Caatinga areas and reinforce the need for management and conservation plans for *P. flavocincta* in priority areas, where the taxon potentially finds stable climatic conditions in past and future scenarios. Protection measures for this taxon are particularly important because this insect contributes to the local flora as it exhibits generalist behavior. Moreover, *P. flavocincta* contributes to local communities as a source of income from honey production, as it is the second most farmed bee species in the state of Rio Grande do Norte (Maia, Jaffe, Carvalho, & Fonseca, 2015) and the third most farmed in the state of Ceará (Felix, 2015).

## CONFLICT OF INTERESTS

The authors have declared that no competing interests exist.

## AUTHOR CONTRIBUTION

**Ulysses Madureira Maia:** Formal analysis (equal); Investigation (lead); Methodology (equal); Visualization (lead); Writing‐original draft (lead); Writing‐review & editing (lead). **Leonardo de Sousa Miranda:** Formal analysis (equal); Investigation (equal); Methodology (equal); Software (equal); Supervision (equal); Validation (equal); Visualization (equal); Writing‐original draft (equal); Writing‐review & editing (equal). **Airton Torres Carvalho:** Investigation (equal); Methodology (equal); Validation (equal); Visualization (equal). **Vera Imperatriz‐Fonseca:** Conceptualization (equal); Project administration (equal); Resources (equal); Supervision (equal). **Guilherme Corrêa de Oliveira:** Investigation (equal); Supervision (equal); Validation (equal); Visualization (equal). **Tereza Cristina Giannini:** Conceptualization (equal); Formal analysis (equal); Funding acquisition (equal); Investigation (equal); Methodology (equal); Project administration (equal); Resources (equal); Supervision (equal); Validation (equal); Visualization (equal); Writing‐original draft (supporting); Writing‐review & editing (supporting).

## Supporting information

Appendix S1

## Data Availability

All relevant data are within the manuscript and its Supporting Information files (Appendix S1).
